# Protective efficacy of a genetically modified attenuated vaccinia virus Tiantan strain against monkeypox virus challenge in a small animal model

**DOI:** 10.1128/jvi.01843-25

**Published:** 2026-01-27

**Authors:** Wenhao Su, Tingting Zhao, Xiuxiu Ren, Shishi Li, Qiufang Huang, Jingjing Liu, Xiaohuan Zhang, Zihao Ge, Jiangbo Wei

**Affiliations:** 1 Weijiangbo Laboratory, National Vaccine & Serum Institute (NVSI) 111611, Beijing, China; 2 State Key Laboratory of Novel Vaccines for Emerging Infectious Diseases, Beijing, China; 3 National Engineering Research Center for Novel Vaccines, Beijing, China; Michigan State University, East Lansing, Michigan, USA

**Keywords:** monkeypox virus, monkeypox vaccine, vaccinia virus Tiantan strain, attenuated vaccinia virus, virulence

## Abstract

Vaccinia virus (VACV) confers cross-protective immunity against monkeypox virus (MPXV), the causative agent of mpox, and has therefore been extensively exploited as a preventive vaccine. VACV Tiantan strain (VTT) is a second-generation smallpox vaccine used in China in the last century, and there are consistent efforts to minimize its virulence and ensure its best safety for potential clinical applications. In this study, an attenuated VACV rVTT△C12K2△A45 was constructed by deletion of gene segments related to virulence genes, host range genes, immune regulatory genes, and other functional genes from the VTT genome by genetic engineering. Attenuation characteristics of rVTT△C12K2△A45 were confirmed by smaller plaque size, lower replication capacity in various mammalian cell lines along with tests for neurotoxicity in mice, and lesion formation on rabbit skin. Immunization in BALB/c mice with rVTT△C12K2△A45 induced both anti-MPXV and anti-VACV neutralizing antibodies. Animals vaccinated with rVTT△C12K2△A45 showed lower MPXV viral loads in the lungs and genital organs compared to the non-immunized mice.

**IMPORTANCE:**

The World Health Organization declared monkeypox a “Public Health Emergency of International Concern” twice, in 2022 and 2024, respectively. Smallpox vaccines have shown efficacy in protecting against monkeypox because of the cross-protective immunity among orthopoxviruses. The vaccinia virus Tiantan strain (VTT) played a critical role in China’s smallpox eradication campaign. Here, we construct an attenuated vaccinia virus by deletion of different ranges of genes in the VTT genome. This attenuated vaccinia virus replicates like its parental VTT strain in production CEF cells but is severely impaired in human-derived cells like 2BS, MRC-5, and WI-38 cells. Meanwhile, this virus shows significantly reduced virulence in small animals. Animals vaccinated with this attenuated vaccinia virus showed lower monkeypox virus (MPXV) viral loads in the lungs and genital organs compared to the non-immunized mice after MPXV challenge. Our data suggest the potential of this genetically engineered VTT strain as a MPXV vaccine candidate.

## INTRODUCTION

Monkeypox is a zoonotic infectious disease caused by the monkeypox virus (MPXV). Since its first discovery in monkeys in 1958, MPXV has been confined to endemic areas in Western and Central Africa, with rare person-to-person transmission within locals and international travelers (1–4). Person-to-person transmission of MPXV has only been recognized as a significant threat after an outbreak in Nigeria in 2018 (5–7). In 2022, a global monkeypox outbreak emerged (8–11). The World Health Organization (WHO) declared monkeypox a “Public Health Emergency of International Concern” twice, in July 2022 and August 2024, respectively (12, 13).

MPXV is a double-stranded DNA virus belonging to the *Orthopoxvirus* genus of the *Poxviridae* family, which also includes several animal poxviruses, vaccinia virus, cowpox virus, and variola virus (14, 15). Vaccinia virus is used as smallpox vaccines and exhibits broad serological cross-reactivity with MPXV (16–19). As of today, two smallpox vaccines, Jynneos and ACAM2000, have been licensed for the prevention of MPXV infection by the US Food and Drug Administration (20, 21). Jynneos is a third-generation smallpox vaccine made from the modified vaccinia Ankara (MVA) strain cultured in primary chicken embryo fibroblast (CEF) cells. It is unable to replicate in most mammalian cells (22, 23), offering a lower risk of serious adverse events than the second-generation smallpox vaccine ACAM2000 and other previous generations of vaccines (24, 25).

The vaccinia virus Tiantan strain (VTT) played a critical role in China’s smallpox eradication campaign. The MPXV membrane proteins (A29L, A35R, M1R, E8L, H3L, and B6R) share more than 95% sequence identity with its VTT counterparts (26). Studies have shown that serum from VTT-immunized mice and rabbits exhibits cross-reactivity with MPXV antigens (27), indicating VTT’s potential as a monkeypox vaccine. However, intracerebral inoculation in murine animals has shown that VTT has certain neurotoxicity, and similar toxicity has been observed in human vaccination, sometimes resulting in various vaccine-related complications (28, 29). Therefore, there is a certain necessity to further enhance the safety of VTT as a potential vaccine. In this study, the virulence, host range, immune regulatory, and other functional genes in the VTT genome were selectively knocked out by genetic engineering methods for attenuation, and the attenuated strain was then verified as potential monkeypox vaccine.

## RESULTS

### Construction and screening of the recombinant vaccinia virus

**Fig 1:**
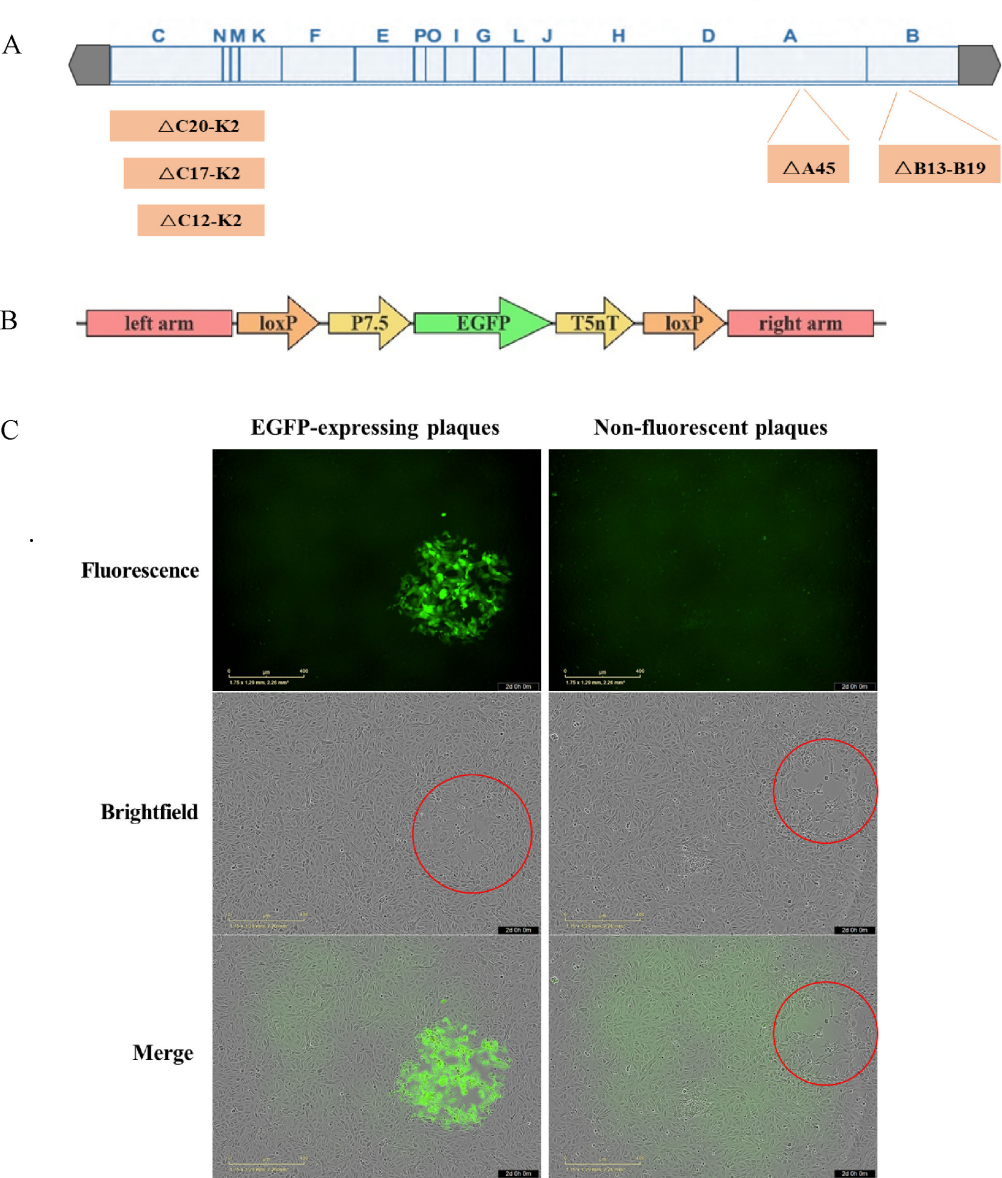
Construction of the recombinant vaccinia virus. (**A**) Schematic representation of the targeted deletion regions in the VTT genome. (**B**) Schematic representation of donor plasmids. (**C**) Vero cells were infected with EGFP-carrying recombinant vaccinia viruses or non-fluorescent viruses and examined for EGFP expression by fluorescence microscopy or plaque formation (red circles) under brightfield microscopy.

Based on CRISPR-Cas9-mediated homologous recombination, the EGFP gene was used as a screening marker to replace the genes C12L-K2L, C17L-K2L, C20L-K2L, B13R-B19R, and A45R in the VTT genome, respectively (Fig. 1A and B), thereby generating the recombinant viruses rVTT△C12K2-EGFP, rVTT△C17K2-EGFP, rVTT△C20K2-EGFP, rVTT△C12K2△B13B19-EGFP, and rVTT△C12K2△A45-EGFP, respectively. All plaques exhibited green fluorescence after six rounds of plaque purification under fluorescence microscopy (Fig. 1C). Sequencing of the genome editing regions confirmed that the targeted deletion regions were successfully replaced by EGFP.

Using the Cre/loxP system, the EGFP genes of rVTT△C12K2-EGFP and rVTT△C12K2△A45-EGFP were knocked out, resulting in recombinant vaccinia viruses rVTT△C12K2 and rVTT△C12K2△A45 without screening marker genes. None of the plaques expressed green fluorescence after three rounds of plaque purification under fluorescence microscope (Fig. 1C). Sequencing results confirmed the deletion of EGFP, with only a fragment of the loxP sequence remaining in the target region.

### rVTT △C12K2-EGFP has lower virulence than rVTT△C17K2-EGFP and rVTT△ C20K2-EGFP

**Fig 2:**
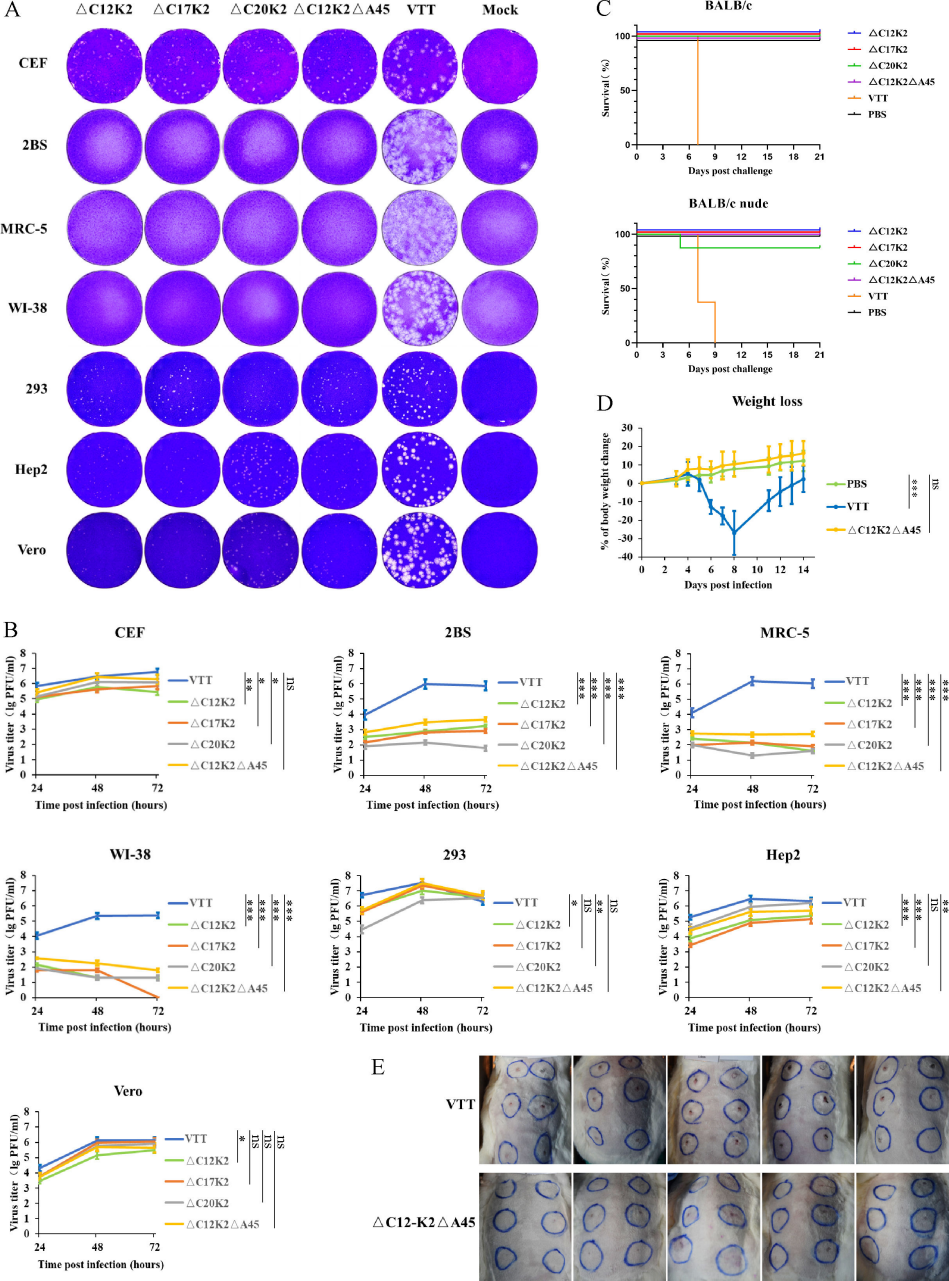
Assessment of attenuation of recombinant vaccinia virus. (**A**) Plaque size phenotype of VTT and recombinant viruses. (**B**) Growth curves of VTT and recombinant viruses. (**C**) Survival of BALB/c (*n* = 10 per group) and BALB/c nude mice (*n* = 10 per group) intracerebrally inoculated with VTT or recombinant viruses. (**D**) Weight changes of BALB/c mice (*n* = 10 per group) intranasally inoculated with VTT or rVTT△C12K2△A45. (**E**) Skin lesions (day 21 post-inoculation) in rabbits (*n* = 5 per group) intradermally inoculated with VTT or rVTT△C12K2△A45. Data were presented as mean ± SD in (**B and D**). The statistical significance was determined by one-way ANOVA in (**B**); two-way ANOVA in (**D**). *P < 0.05, **P < 0.01, and ***P < 0.001 were considered statistically significant, ns (non-significant) for *P* ≥ 0.05.

The virulence of rVTT△C12K2-EGFP, rVTT△C17K2-EGFP, and rVTT△C20K2-EGFP was significantly reduced as compared to their parental VTT strain, evidenced by the observation that plaque size of all three recombinant vaccinia viruses was obviously smaller than those of the parental VTT strain on various cell types (Fig. 2A). The peak titers of these three recombinant viruses in the production CEF cells were comparable to those of VTT, but they could not replicate effectively in human-derived cells 2BS, MRC-5, and WI-38 (Fig. 2B). In the BALB/c mice neurovirulence test, no death was observed in groups infected with the recombinant viruses at a dose of 5 × 10^5^ PFU, whereas all mice died at a VTT dose of 500 PFU (Fig. 2C).

Among the three recombinant vaccinia viruses, rVTT△C12K2-EGFP formed smaller plaques on HEK 293, Hep2, and Vero cells than rVTT△C17K2-EGFP and rVTT△C20K2-EGFP. In the BALB/c nude mice neurovirulence test, animal death was observed in the rVTT△C20K2-EGFP infection group. Based on the above results, rVTT△C12K2-EGFP was chosen as a preferable candidate for later researches.

### Deletion of B13R-B19R in the rVTT△C12K2 genome impairs virus production in CEF cells

The B13R-B19R region was deleted in the rVTT△C12K2 genome to generate rVTT△C12K2△B13B19-EGFP. To compare these two virus strains, CEF cells were infected with the two viruses separately at a multiplicity of infection (MOI) of 0.01. After incubation at 37°C for 3 days, virus titers were determined using Vero cells. Plaque size of rVTT△C12K2△B13B19-EGFP was smaller than that of rVTT△C12K2, and the titer of different clones of rVTT△C12K2△B13B19-EGFP was 100–1,000 times lower than that of rVTT-C12-K2.

### Deletion of A45R in the rVTT△C12K2 genome increases IFN-γ secreting splenocytes

**Fig 3:**
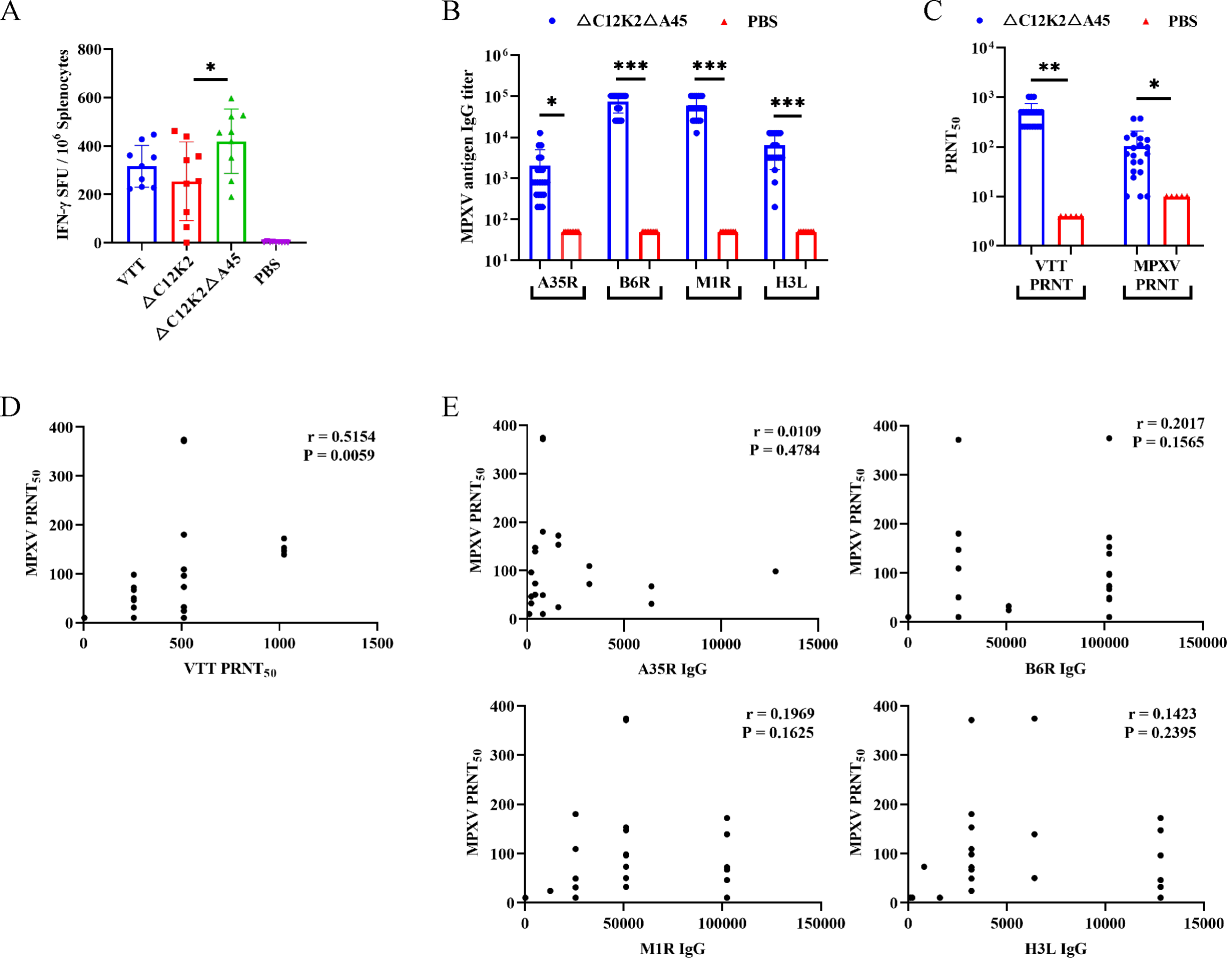
Immune responses in vaccinated BALB/c mice. (**A**) IFN-γ response induced in mice (*n* = 9 per group) by VTT or recombinant viruses. (**B**) MPXV antigen-specific binding antibody induced in mice (*n* = 20) by rVTT△C12-K2△A45. (**C**) VTT and MPXV-neutralizing antibody induced in mice (*n* = 20) by rVTT△C12-K2△A45. (**D**) Correlation between MPXV-neutralizing antibody and VTT-neutralizing antibody. (**E**) Correlation between MPXV-neutralizing antibody and binding antibody. Data were presented as mean ± SD in (**A–C**). The statistical significance was determined by one-way ANOVA in (**A**); Student’s *t*-test in (**B and C**). *P < 0.05, **P < 0.01, and ***P < 0.001 were considered statistically significant. Pearson correlation coefficients (*r*) were used to analyze correlation between VTT-neutralizing antibody titer and MPXV-neutralizing antibody titer (**D**) and MPXV-binding antibody titer and MPXV-neutralizing antibody titer (**E**).

Six- to 8-week-old BALB/c mice were immunized intramuscularly with two doses of 1 × 10^6^ PFU rVTT△C12K2, rVTT△C12-K2△A45, or VTT on days 0 and 28. Phosphate-buffered saline (PBS) was used in a negative control. On day 35, spleens were collected and analyzed by IFN-γ ELISpot assay. VTT-specific IFN-γ-secreting splenocytes induced by rVTT△C12-K2△A45 increased significantly in comparison to those induced by rVTT△C12K2 (Fig. 3A). This IFN-γ level was also higher than those induced by VTT, although the difference was not statistically significant.

### Assessment of attenuation of rVTT△C12K2△A45

The plaques formed by rVTT△C12K2△A45 in various cell types were significantly smaller than those formed by the parent virus VTT (Fig. 2A). Similar to its rVTT△C12K2 origin as previously described, the peak titers of rVTT△C12K2△A45 in production CEF cells were comparable to those of VTT, but rVTT△C12K2△A45 could not replicate effectively in human-derived cells 2BS, MRC-5, and WI-38 (Fig. 2B). In BALB/c and BALB/c nude mice neurovirulence tests, no deaths occurred in groups infected with the rVTT△C12K2△A45 at a dose of 5 × 10^5^ PFU, in contrast to a total mortality in the VTT group at a dose of 500 PFU (Fig. 2C). Following intranasal infection with rVTT△C12K2△A45, there was no observed weight loss in BALB/c mice, whereas VTT-infected mice experienced a weight loss that peaked at 29% on day 8 (Fig. 2D). In the virulence test on rabbit back skin, the average diameter of lesions caused by rVTT△C12K2△A45 was smaller than those caused by VTT (Fig. 2E).

### Humoral immune responses elicited by rVTT△C12K2△A45

Six- to 8-week-old BALB/c mice were immunized intramuscularly with two doses of 1 × 10^8^ PFU rVTT△C12K2△A45 on days 0 and 28. PBS was used in a negative control. Fourteen days after the second dose of vaccination, serum was collected for antibody testing. Specific antibodies against MPXV antigens (A35R, B6R, M1R, and H3L) were detected by enzyme-linked immunosorbent assay (ELISA) (Fig. 3B), and neutralizing antibodies against VTT and MPXV were tested by PRNT (Fig. 3C). Correlation analysis was performed between binding antibody, VTT-neutralizing antibody, and MPXV-neutralizing antibody. A moderate correlation was found between MPXV-neutralizing antibody titer and VTT-neutralizing antibody titer (*r* = 0.5154, Fig. 3D), whereas the correlation between MPXV-neutralizing antibody titer and binding antibody titer was weak (Fig. 3E)

### rVTT△C12K2△A45 immunization protects mice from VTT and MPXV challenge

**Fig 4:**
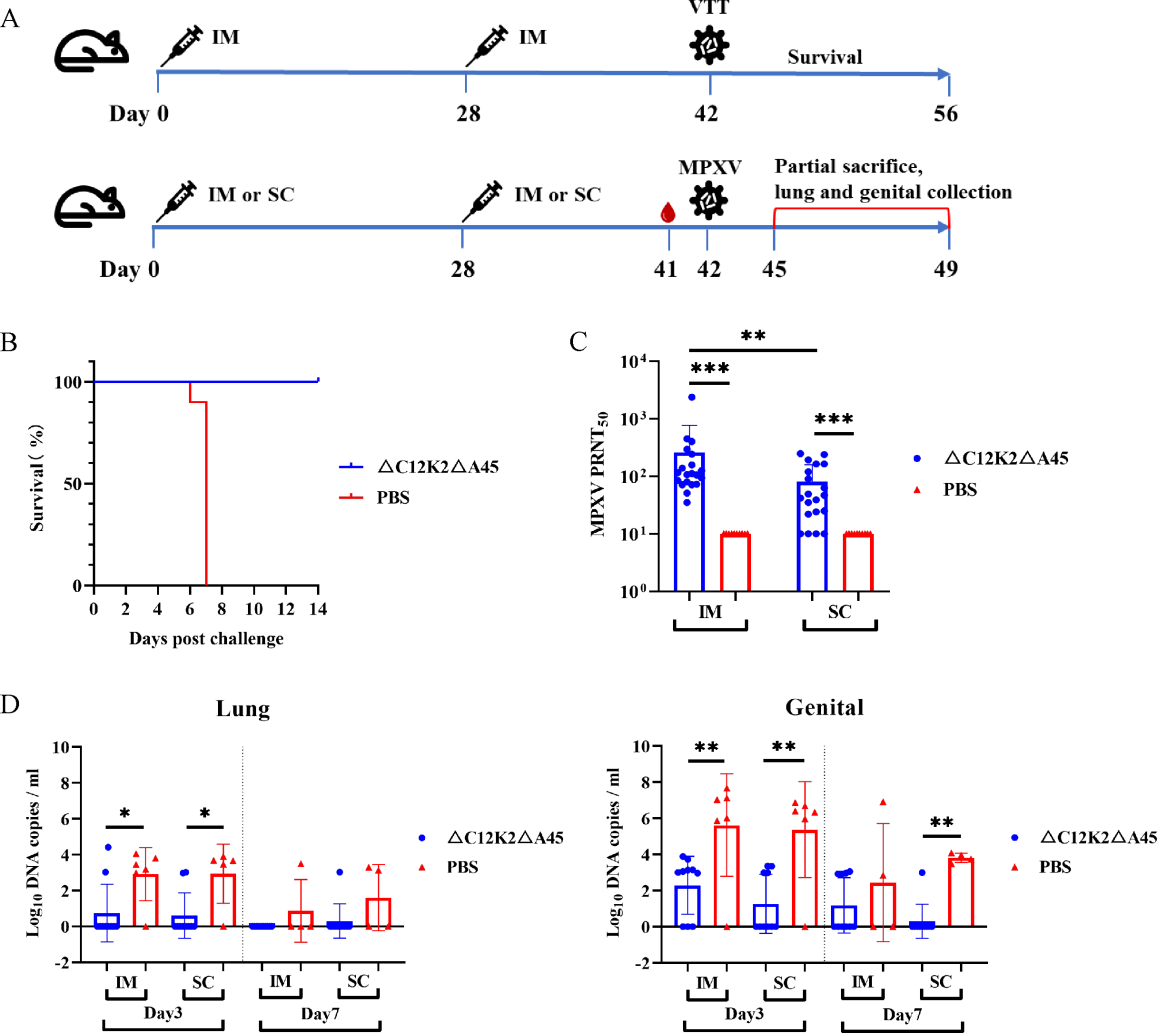
Protective efficacy against VTT or MPXV challenge in vaccinated BALB/c mice. (**A**) Study design to evaluate protective efficacy of rVTT△C12K2△A45 against VTT or MPXV challenge. (**B**) Protective efficacy of rVTT△C12K2△A45 against lethal VTT challenge. (**C**) MPXV-neutralizing antibody induced by rVTT△C12-K2△A45 in intramuscularly (IM) or subcutaneously (SC) immunized mice (*n* = 20 for vaccine-immunized mice, *n* = 10 for PBS control group). (**D**) Viral loads in mice (vaccine-immunized mice: *n* = 10 at day 3 and day 7; PBS control group: *n* = 6 at day 3 and *n* = 4 at day 7) lungs and genitals after MPXV challenge. Data were presented as mean ± SD in (**C and D**), the statistical significance was determined by Student’s *t*-test. *P < 0.05, **P < 0.01, and ***P < 0.001 were considered statistically significant.

To assess protective efficacy of rVTT△C12K2△A45 against VTT challenge, mice were immunized intramuscularly and later challenged intracranially with a lethal dose of VTT (Fig. 4A). All immunized mice survived; by contrast, all mice died in the negative control group (Fig. 4B).

In a MPXV-infected mouse model (Fig. 4A), the protective efficacy was evaluated by intramuscular immunization and subcutaneous immunization. Serum was collected on the day before the challenge to detect MPXV-neutralizing antibody. The antibody level via intramuscular route (geometric mean titer [GMT] = 136.52) was higher than that via subcutaneous route (GMT = 45.54) (Fig. 4C). On days 3 and 7 post-challenge, lungs and genital tissues were collected to determine viral loads. Lower MPXV viral loads in mouse lungs and genital organs were detected than the PBS control group, regardless of the route of immunization (Fig. 4D).

## DISCUSSION

The WHO declared the global eradication of smallpox in 1980. Various strains of vaccinia virus have been developed as vaccines to prevent smallpox during the eradication era, including New York City Board of Health (NYCBH)/Dryvax in the United States, Lister in Europe, EM-63 in Russia, and VTT in China (30, 31). In 1926, the VTT strain was isolated from a smallpox patient at Beijing’s Temple of Heaven by Mr. Qi Changqing, an employee in the Chinese National Epidemic Prevention Bureau. Then the virus had an attenuation history of passages on the skin of monkeys, rabbits, and cows (32). Based upon the viral genome sequencing done to date, VTT is certainly a vaccinia virus (33). Phylogenetic analysis using the alignments of the conserved central region F9L-to-A24R (32) and C7L-to-B16R (33) (orthologous region in Copenhagen strain) showed that VTT was most similar to Copenhagen/Lister lineage and distinct from the Dryvax lineage. We aligned full genome between VTT and other vaccinia virus strains, including Copenhagen, Lister, and Dryvax (Fig. S1A and B). Genetic similarities between VTT and other vaccinia virus strains varied from 98.12% to 98.59%, and VTT had the highest identity scores with Copenhagen. Among these analyzed viruses, a higher accumulation of differences was observed in the terminal regions of the genome which are prone to high frequencies of mutation. The central region in the genome seems to be conserved between the different viruses. It is worth noting that in the central region, there is a 4.1 kbp region (bp 134827–138927) in VTT encoding cowpox A-type inclusion protein that had orthologous region in Lister and Dryvax but was absent in Copenhagen.

Variola virus, MPXV, and vaccinia virus all belong to the genus *Orthopoxvirus*, offering a rationale for current MPXV vaccine development. Second-generation smallpox vaccine ACAM2000 and third-generation smallpox vaccine Jynneos have been approved for use in humans against monkeypox. However, replication-competent ACAM2000 may cause serious adverse reactions and is contraindicated in immunocompromised people (25). Jynneos, a non-replicating modified vaccinia virus Ankara (MVA) vaccine attenuated by serial passage of chorioallantois vaccinia virus Ankara over 570 times on CEF cells, had a better safety profile than ACAM2000. MVA acquired six large genomic deletions during serial passage (34), all these major deletions occurred in the genome ends that showed high genetic divergence among orthopoxviruses. A total of 31 open reading frames (ORFs) in the 6 major deletions were lost or truncated (Table S1).

In this study, we constructed an attenuated vaccinia virus strain rVTT△C12K2△A45 based on VTT. A total of 18 ORFs (C12L-K2L) were deleted in the VTT left terminal region, and another ORF (A45R) was deleted in the right terminal region by CRISPR-Cas9-mediated homologous recombination. A comparison (Table S1) of rVTT△C12K2△A45 with MVA revealed that both viruses shared common deleted regions: C5L-N1L and M1L-K1L. C12L-C6L region deleted in rVTT△C12K2△A45, encoding two host range proteins CP77 (C12L and C11L) and C7L (34), was present in MVA. However, deletion of the major host range genes (CP77, C7L, and K1L) in the significantly attenuated rVTT△C12K2△A45 did not lead to the MVA phenotype of highly restricted host range, the VTT mutant strain still replicated effectively in some mammalian cells such as 293 and Vero cells, suggesting that deletion of C12L-K2L region in VTT is not sufficient to reproduce the MVA phenotype. However, to some extent, the unique profile of rVTT△C12K2△A45 may make it a better MPXV vaccine, as it acquired the ability to replicate in Vero cells considered as the most widely used continuous cell line for vaccine production and also used for ACAM2000 manufacture (35), whereas embryonated egg-based production limits manufacturing capacity of MVA. The A35R gene in MVA, an immune regulatory gene and its expression products have an inhibitory effect on MHCII antigen presentation (36), is the homolog of the A45R gene in VTT. Deletion of the A45R gene in rVTT△C12K2△A45 resulted in increased IFN-γ secreting splenocytes. We also knocked out the B13R-B19R region encoding serine proteinase inhibitor (B13R, B14R), interleukin-1β receptor (B15R, B16R), and type I interferon receptor (B18R, B19R) (37) in rVTT△C12K2△B13B19, and the virulence of the resulted VTT mutant was further reduced; however, its replication was severely inhibited in production cell CEF.

Vaccination with VTT and VTT-based attenuated virus was effective in stimulating neutralizing antibodies against MPXV and VACV in mouse and rhesus monkey models (38–40). However, a study showed that VTT is inefficient in eliciting cross-reactive immunity against the MPXV (41), probably due to the intraperitoneal route of vaccination used in the study inducing low levels of neutralizing antibodies. Immunization routes profoundly affect vaccine immunogenicity. VTT induced different levels of neutralizing antibodies via various routes of vaccination; anti-VTT-neutralizing antibody titer induced by oral vaccination was 5.5-fold higher than that induced by intramuscular vaccination and 74-fold higher than that induced by subcutaneous vaccination (42), as we reported above that intramuscular injection of rVTT△C12K2△A45 induced about three times higher MPXV-neutralizing antibody GMT than that induced by subcutaneous injection.

We first examined whether rVTT△C12K2△A45 stimulated cross-reactive immune responses to MPXV antigen. MPXV exists in two infectious forms: extracellular enveloped virus (EEV) and intracellular mature virion (IMV), which have different outer proteins (43). Two EEV antigens (A35R and B6R) and two IMV antigens (M1R and H3L) were selected to detect MPXV-specific binding antibodies. rVTT△C12K2△A45 induced robust responses to B6R and M1R but relatively weak responses to A35R and H3L. By contrast, rVTT△C12K2△A45 induced anti-M1R and anti-H3L IgG titers similar in magnitude to the MVA response, lower anti-A35R IgG titers, and higher anti-B6R IgG titers (44). A recent study (45) indicated that anti-M1R and anti-B6R binding antibodies correlated with protection for MPXV vaccine. This suggests that rVTT△C12K2△A45 may confer improved protective efficacy against MPXV. In addition, MPXV cross-reactive neutralizing antibody responses in mice were measured, antibody levels induced by two doses of intramuscularly administered rVTT△C12K2△A45 were comparable to those induced by MVA with same vaccination route (44, 46). However, subcutaneously administered rVTT△C12K2△A45 induced lower anti-MPXV and anti-VTT-neutralizing antibody than that induced by intramuscular administration, while subcutaneous immunization of MVA induced higher serum neutralizing responses (47, 48). It is reported that intranasal or intraoral vaccination of VTT or VTT-vectored vaccine induced substantially higher neutralizing antibody responses than that induced via the intramuscular or subcutaneous route (42) and to increase levels of anti-MPXV-neutralizing antibody induced by rVTT△C12K2△A45, intranasal or intraoral vaccination route could be used in the future study.

Then we evaluated the protective effect of rVTT△C12K2△A45 against VTT and MPXV infection in the challenge-protection experiment. The vaccine provided 100% protection for mice against lethal intracerebral challenge. Transmission of MPXV during the 2022 global outbreak involved men-men sexual activity and mucosal exposure, and there was increased sexual transmission caused by the more pathogenic clade I MPXV strains (49). Accordingly, we assessed the protective effect of the vaccine against MPXV infection by detecting viral loads in the lungs and genitals in the MPXV-infected mouse model. rVTT△C12K2△A45 provided robust protection comparable to MVA, and lower MPXV viral loads were detected in the mouse lungs and genitals in both subcutaneous and intramuscular immunization groups than the control. Some animals (2/10) vaccinated with rVTT△C12K2△A45 showed detectable viral loads in the lungs; in contrast, 2/8 mice in MVA vaccinated animals (46). A study showed a single dose of replication-competent ACAM2000 provided complete protection, vaccinated macaques had no detectable viral loads in ACAM2000 group, whereas MVA provided robust but incomplete protection (45), suggesting that attenuation and immunogenicity should be balanced in genetically engineered VACV-based MPXV vaccines. Although the MPXV-neutralizing antibody GMT induced by intramuscular injection was three times higher than that induced by subcutaneous injection, subcutaneous injection demonstrated faster viral clearance in the genitalia, implying that other immune responses play important roles beyond neutralizing antibodies in preventing MPXV infection, such as non-neutralizing antibody functions (45, 46) and cellular immune responses (46, 50). MPXV vaccine protective immune correlates remain unvalidated, and more comprehensive and larger-scale studies are needed to confirm these findings.

In summary, we constructed an attenuated vaccinia virus strain rVTT△C12K2△A45 based on VTT, with lower virulence in different cell lines and small animals than its parent VTT strain. Immunization in mice induced neutralizing antibodies against VTT and protected them from lethal VTT challenge. This strain also elicited MPXV antigen-specific binding antibodies and neutralizing antibodies against MPXV in mice when tested as a potential MPXV vaccine, and vaccinated mice manifested significantly fewer MPXV viral loads in the lungs and genitals. These data suggest the potential of this genetically engineered VTT strain as a vaccine candidate that targets MPXV.

## MATERIALS AND METHODS

### Cells, viruses, and animals

HEK 293, Vero, and Hep2 cells were cultured in Dulbecco’s modified Eagle medium (DMEM, Gibco), supplemented with 10% fetal bovine serum (FBS, Gibco) and 1% penicillin-streptomycin (PS, Hyclone). CEF, 2BS, MRC-5, and WI-38 cells were cultured in modified essential medium (MEM, Gibco), containing 10% FBS and 1% PS.

VTT was preserved in National Vaccine and Serum Institute and was propagated in CEF cells. MPXV clade II was preserved in Wuhan Institute of Biological Products, and all experiments involving infectious MPXV were performed in biosafety level 3 (BSL-3) facilities.

Specific-pathogen-free (SPF) BALB/c mice and SPF BALB/c nude mice were purchased from Beijing Vital River Laboratory Animal Technology. New Zealand rabbits were purchased from Qingdao Kangda Aibo Biotechnology.

### Plasmid construction

Based on the VTT genome (GenBank: AF095689.1), homologous arms flanking the C12L-K2L (bp 11762–25450), C17L-K2L (bp 7504–25450), C20L-K2L (bp 4274–25450), B13R-B19R (bp 173213–179856), and A45R (bp 144139–144669) sequences were designed. loxP sequence, P7.5 promoter, EGFP gene, T5nT termination signal, and loxP sequence were sequentially inserted between the homologous arms (Fig. 1B). These constructs were synthesized and ligated into the pUC57 vector by GenScript Biotechnology Corporation, resulting in HDR (homology directed repair) donor plasmids pUC57-C12K2, pUC57-C17K2, pUC57-C20K2, pUC57-B13B19, and pUC57-A45.

The gRNAs were designed and selected using online tools (https://www.zlab.bio/resources). The gRNA expression plasmids were constructed by annealing two complementary oligos from each target sequence and ligating the resulting dsDNA fragment into the BbsI site of the CRISPR/Cas9 system vector pX459.

The Cre gene was synthesized and ligated into the pcDNA3.1(+) by Tsingke Biotechnology Corporation to obtain the plasmid pcDNA3.1(+)-cre.

### Replacement of target genes to be deleted in the VTT genome with EGFP gene

HEK 293 cells were seeded at 1.6 × 10^6^ cells/well in six-well plates and cultured overnight. Cells were transfected with donor plasmid and gRNA expression plasmid (3 μg each) using calcium phosphate transfection reagent. One day later, 3 × 10^4^ PFU of VTT or recombinant virus rVTT△C12K2 was added into each well. After 3 days, cells were subjected to three freeze-thaw cycles to release the viruses. Harvested viruses were used to infect CEF cells, and cultures were maintained in MEM containing 1% methylcellulose for 2–3 days. Green fluorescent plaques were marked under a fluorescence microscope and picked out. Recombinant vaccinia viruses rVTT△C12K2-EGFP, rVTT△C17K2-EGFP, rVTT△C20K2-EGFP, rVTT△C12K2△B13B19-EGFP, and rVTT△C12K2△A45-EGFP were obtained after six rounds of plaque purification.

### Removal of the EGFP gene from recombinant vaccinia virus using Cre-loxP

Similarly, HEK 293 cells were transfected with 3 μg pcDNA3.1(+)-cre. One day later, 3 × 10^4^ PFU of EGFP-carrying recombinant virus rVTT△C12K2-EGFP or rVTT△C12K2△A45-EGFP was added to each well. After 3 days, cells were subjected to three freeze-thaw cycles to release the viruses. Harvested viruses were used to infect CEF cells, and cultures were maintained in MEM containing 1% methylcellulose for 2–3 days. Non-green fluorescent plaques were marked under a fluorescence microscope and picked out, and plaque purification was repeated three times to obtain recombinant vaccinia viruses rVTT△C12K2 and rVTT△C12K2△A45.

### Virus titration

Virus titers were determined by plaque assay. The viruses were 10-fold serially diluted in DMEM containing 10% FBS and added to the monolayers of Vero cells. After absorption at 37°C for 1 h, DMEM containing 1% methylcellulose was added. After 4–5 days of incubation at 37℃, cells were stained with crystal violet, and plaques were counted. Virus titers were defined in terms of PFU/mL.

### Plaque formation of virus in different cell lines

Monolayers of CEF, 2BS, MRC-5, WI-38, HEK 293, Hep2, and Vero cells grown in six-well plates were infected with 100 PFU of recombinant vaccinia viruses or VTT. After absorption at 37°C for 1 h, DMEM containing 1% methylcellulose was added. After 2–4 days, cells were stained with crystal violet. The diameter of viral plaques was analyzed in S6 Universal Analyzer (CTL).

### Viral growth kinetics in different cell lines

Monolayers of CEF, 2BS, MRC-5, WI-38, HEK 293, Hep2, and Vero cells grown in six-well plates were infected with either recombinant vaccinia viruses or VTT at a MOI of 0.01. Culture supernatants and cells were collected at 24, 48, and 72 h post-infection, subjected to three freeze-thaw cycles. Titers were calculated by plaque assay to generate growth curves.

### Weight changes in mice after infection

Five-week-old BALB/c mice were intranasally inoculated with 2 × 10^5^ PFU/20 μL recombinant vaccinia viruses or 2 × 10^3^ PFU/20 μL VTT. PBS was used in the control group. Mice were observed and weighed for 14 days post-infection.

### Neurotoxicity in mice

Three-week-old BALB/c mice and BALB/c nude mice were inoculated intracerebrally with 5 × 10^5^ PFU/20 μL recombinant vaccinia viruses or 5 × 10^2^ PFU/20 μL VTT. PBS was used in the control group. Mice were monitored for 21 days post-infection, and animal death was recorded daily.

### Skin lesions in rabbits

New Zealand rabbits weighing 2.5–3 kg were shaved on the back and intradermally inoculated into six sites with 1 × 10^7^ PFU/100 μL recombinant vaccinia viruses or VTT per site. PBS was used in the control group. Rabbit skin was observed daily, and the diameters of lesions were measured daily for 21 consecutive days.

### IFN-γ ELISpot assay

Splenocytes of immunized mice were harvested, and IFN-γ secretion was evaluated using mouse IFN-γ ELISpot kits (Mabtech) according to the manufacturer’s protocol. Briefly, 1 × 10^6^ splenocytes were added to the pre-coated ELISpot plates, followed by a stimulation with VTT (MOI = 0.1) at 37°C for 16–20 h. Plates were then washed and incubated with biotinylated detection antibody for 2 h at room temperature. Plates were washed, incubated with Streptavidin-ALP for 1 h at room temperature, and then washed again. BCIP/NBT substrate was added to the plates for spot coloration. The ELISpot plates were analyzed in S6 Universal Analyzer (CTL).

### VTT-neutralizing antibody assay

VTT-neutralizing antibody titers were determined by 50% plaque reduction neutralization test (PRNT50). Briefly, serum samples were twofold serially diluted and then mixed with an equal volume of VTT (final virus concentration: 100 PFU/0.1 mL). After 1 h of neutralization at 37℃, 0.1 mL of the serum-virus mixture aliquot was transferred into 24-well plates pre-seeded with Vero cells. Simultaneously, 100 PFU/0.1 mL VTT was used to infect Vero cells as a virus control. After absorption at 37°C for 1 h, DMEM containing 1% methylcellulose was added to the plates. Following 2 days of incubation at 37℃, cells were stained with crystal violet. The neutralizing antibody titer was defined as the highest dilution of the serum that reduced the formation of plaques by 50% in comparison with the plaque number in virus controls.

### MPXV antigen-specific binding antibody assay

MPXV antigen-specific IgG antibody titers were analyzed by the ELISA. Briefly, ELISA plates were coated with 1 μg/mL MPXV antigens A35R, B6R, M1R, or H3L (Sino Biological) and incubated at 37℃ for 1 h. After washing with PBS containing 0.1% Tween-20 (PBST) and blocking with 1% bovine serum albumin, twofold serially diluted serum was added to the wells and incubated at 37℃ for 1 h. Plates were then washed and supplied with horseradish peroxidase (HRP)-conjugated goat anti-mouse IgG (Sino Biological, dilution 1:10,000). After 1 h at 37℃, plates were washed again, and 3,3',5,5'-tetramethylbenzidine (TMB) substrate was added to the wells. After incubation for 10–15 minutes, stop solution was added to the wells, and absorbance values at 450 nm were measured. The antibody titer was defined as the highest dilution of the serum that reaches 2.1 times the mean OD_450_ of the negative serum.

### MPXV-neutralizing antibody assay

MPXV-neutralizing antibody titers were determined by PRNT50. Briefly, serum samples were fourfold serially diluted and then mixed with an equal volume of MPXV (final virus concentration: 150 PFU/0.5 mL). After 1 h of neutralization at 37℃, an aliquot of 0.5 mL of the serum-virus mixture was transferred into 12-well plates pre-seeded with Vero cells. Simultaneously, 150 PFU/0.5 mL MPXV was used to infect Vero cells as a virus control. After absorption at 37°C for 1 h, DMEM containing 0.9% methylcellulose was added to the plates. After 5 days of incubation at 37℃, cells were stained with crystal violet. The neutralizing antibody titer was defined as the highest dilution of the serum that reduced the formation of plaques by 50% in comparison with the plaque number in virus controls.

### VTT challenge

Six- to 8-week-old BALB/c mice were intramuscularly immunized with two doses of 1 × 10^8^ PFU rVTT△C12K2△A45 on days 0 and 28. PBS was used in a negative control. On day 42, mice were intracranially challenged with VTT at a dose of 6.4 × 10^3^ PFU (20 LD50). After the challenge, the mortality was recorded daily for 14 days.

### MPXV challenge

Six- to 8-week-old BALB/c mice were immunized intramuscularly or subcutaneously with 1 × 10^8^ PFU rVTT△C12K2△A45 on days 0 and 28. PBS was used in a negative control. On day 42, mice were challenged intraperitoneally with 1 × 10^7^ PFU MPXV. One day before challenge, serum samples were collected for MPXV-neutralizing antibody testing. On days 3 and 7 post-challenge, lung and genital tissues were collected to assess viral loads.

### Statistical analysis

Statistical differences between two or multiple groups were analyzed by Student’s *t*-test or one-way analysis of variance (ANOVA). *P < 0.05, **P < 0.01, and ***P < 0.001 were considered statistically significant, non-significant for *P* ≥ 0.05. Pearson correlation coefficients (*r*) were used to analyze correlation between binding antibody, VTT-neutralizing antibody, and MPXV-neutralizing antibody.

## Data Availability

All data are available upon request to the corresponding author.

## SUPPLEMENTAL MATERIAL

Supplemental material jvi.01843-25-s0001.docx Fig. S1 and Table S1.

ASM does not own the copyrights to Supplemental Material that may be linked to, or accessed through, an article. The authors have granted ASM a non-exclusive, world-wide license to publish the Supplemental Material files. Please contact the corresponding author directly for reuse.
