# Supplementary material for: Protective efficacy of a genetically modified attenuated vaccinia virus Tiantan strain against monkeypox virus challenge in a small animal model
Source: J Virol. 2026 Jan 27;100(2):e01843-25. doi: 10.1128/jvi.01843-25 (PMC12911895; doi:10.1128/jvi.01843-25)
Supplement: Supplemental material — Fig. S1 and Table S1. [file jvi.01843-25-s0001.docx]

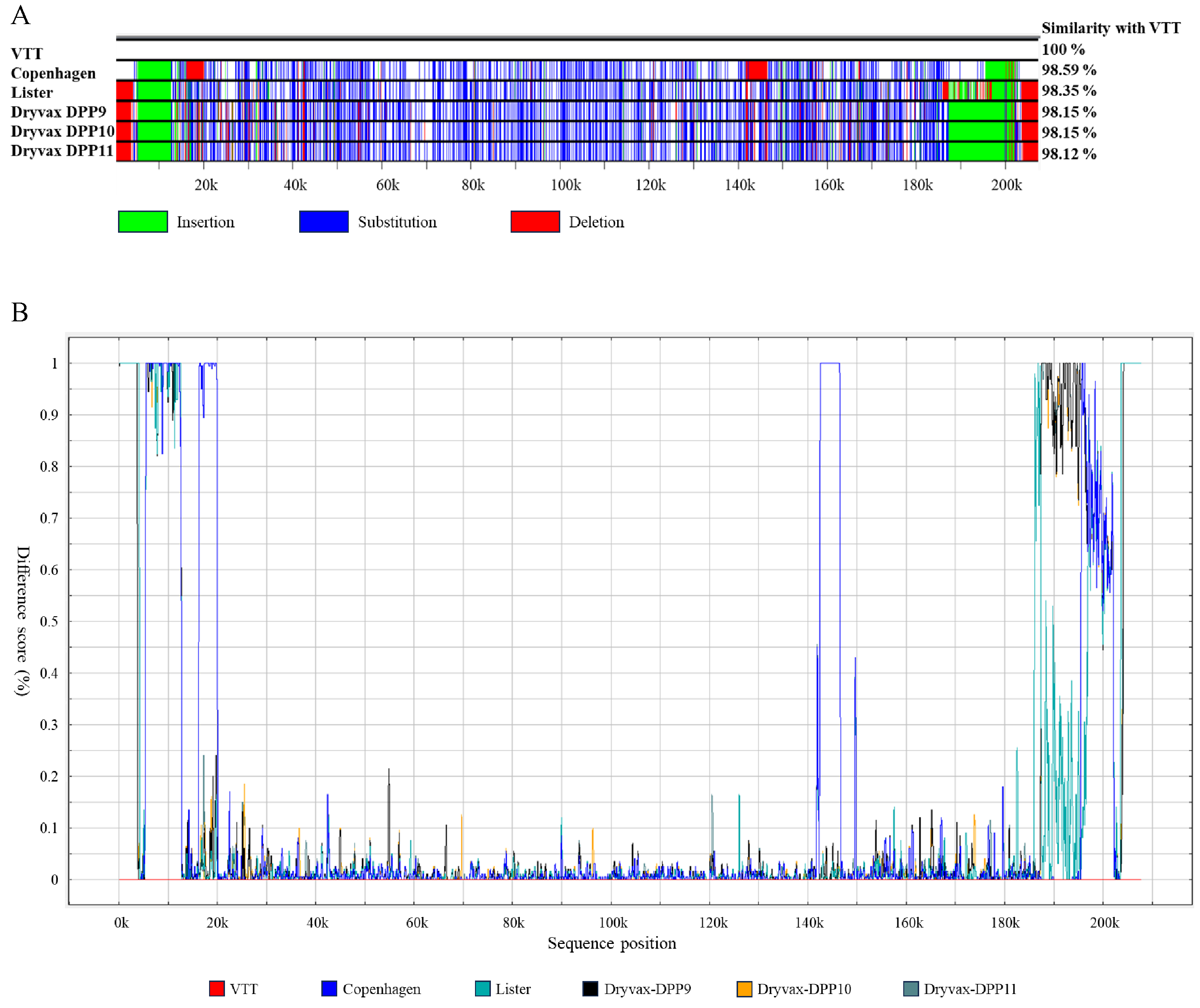


FIG S1 The full genomes of VTT (GenBank: AF095689.1), Copenhagen (M35027.1), Lister (DQ121394.1) and Dryvax clones (DPP9: JN654976.1, DPP10: JN654977.1, and DPP11: JN654978.1) were aligned by Base-by-Base software. (A) Graphical map of genetic differences (insertions, SNPs, and deletions) in relation to the genome of VTT. The similarity (%) of each vaccinia virus strain with VTT is displayed. (B) Differences score per site of Copenhagen, Lister and Dryvax clones in relation to VTT along the genome.

TABLE S1 Comparison of gene deletions in MVA and rVTT△C12K2△A45

| MVA^a^ | rVTT△C12K2△A45 | Orthologues in Copenhagen |
| --- | --- | --- |
| CVA008 |  | C16L |
| CVA009 |  | C15L |
| CVA010 |  | C12L |
|  | C12L | CPXV C9L^b^ |
|  | C11L | CPXV C9L^b^ |
|  | C10L | CPXV C10L^b^ |
|  | C9L | C9L |
|  | C8L | C8L |
|  | C7L | C7L |
|  | C6L | C6L |
| CVA028 | C5L | C5L |
| CVA029 | C4L | C4L |
| CVA030 | C3L | C3L |
| CVA031 | C2L | C2L |
| CVA032 | C1L | C1L |
| CVA033 | N1L | N1L |
|  | N2L | N2L |
| CVA035 | M1L | M1L |
| CVA036 |  | M1L |
| CVA037 | M2L | M2L |
| CVA038 | K1L | K1L |
|  | K2L | K1L |
| CVA153 |  | A26L |
| CVA154 |  | CPXV A26L^b^ |
| CVA155 |  | CPXV A26L^b^ |
| CVA156 |  | A26L |
|  | A45R | A35R |
| CVA186 |  | A51R |
| CVA187 |  | A52R |
| CVA188 |  | A53R |
| CVA189 |  | A55R |
| CVA190 |  | A55R |
| CVA212 |  | B20R |
| CVA213 |  | CPXV B18R^b^ |
| CVA214 |  | CPXV C8L^b^ |
| CVA215 |  | CPXV C7R^b^ |
| CVA216 |  | CPXV C7R^b^ |
| CVA217 |  | CPXV C7R^b^ |
| CVA218 |  | C10L |
| CVA219 |  | C11R |
| CVA220 |  | C12L |

^a^ Genes deleted or truncated in the six major deletions of MVA (U94848.1) in relation to CVA (AM501482.1).

^b^ No homologues exist in Copenhagen, orthologues from cowpox virus (CPXV: X94355.2).
